# Sustained complete response to first-line immunochemotherapy for highly aggressive TP53/MDM2-mutated upper tract urothelial carcinoma with ERBB2 mutations, luminal immune-infiltrated contexture, and non-mesenchymal state: a case report and literature review

**DOI:** 10.3389/fonc.2023.1119343

**Published:** 2023-06-23

**Authors:** Tianyuan Xu, Hanxu Guo, Jun Xie, Yanyan He, Jianping Che, Bo Peng, Bin Yang, Xudong Yao

**Affiliations:** ^1^ Department of Urology, Shanghai Tenth People’s Hospital, Tongji University, Shanghai, China; ^2^ Institue of Urinary Oncology, Tongji University School of Medicine, Shanghai, China; ^3^ Department of Urology, Shanghai Tenth People’s Hospital, Shanghai Clinical College, Anhui Medical University, Shanghai, China; ^4^ Department of Pathology, Shanghai Tenth People’s Hospital, Tongji University, Shanghai, China

**Keywords:** upper tract urothelial carcinoma, immunochemotherapy, TP53/MDM2, ErbB2, luminal-infiltrated, circulating tumor DNA (ctDNA), case report, sintilimab

## Abstract

**Background:**

Upper tract urothelial carcinoma (UTUC) is a rare malignancy. The management of metastatic or unresectable UTUC is mainly based on evidence extrapolated from histologically homologous bladder cancer, including platinum-based chemotherapy and immune checkpoint inhibitor alone, whereas UTUC exhibits more invasiveness, worse prognosis, and comparatively inferior response to treatments. First-line immunochemotherapy regimens have been attempted in clinical trials for unselected naïve-treated cases, but their efficacies relative to standard chemo- or immuno-monotherapy still remain controversial. Here, we present a case of highly aggressive UTUC for whom comprehensive genetic and phenotypic signatures predicted sustained complete response to first-line immunochemotherapy.

**Case presentation:**

A 50-year-old man received retroperitoneoscopic nephroureterectomy and regional lymphadenectomy for high-risk locally advanced UTUC. Postoperatively, he developed rapid progression of residual unresectable metastatic lymph nodes. Pathologic analysis and next-generation sequencing classified the tumor as highly aggressive TP53/MDM2-mutated subtype with features more than expression of programmed death ligand-1, including ERBB2 mutations, luminal immune-infiltrated contexture, and non-mesenchymal state. Immunochemotherapy combining gemcitabine, carboplatin, and off-label programmed death-1 inhibitor sintilimab was initiated, and sintilimab monotherapy was maintained up to 1 year. Retroperitoneal lymphatic metastases gradually regressed to complete response. Blood-based analyses were performed longitudinally for serum tumor markers, inflammatory parameters, peripheral immune cells, and circulating tumor DNA (ctDNA) profiling. The ctDNA kinetics of tumor mutation burden and mean variant allele frequency accurately predicted postoperative progression and sustained response to the following immunochemotherapy, which were mirrored by dynamic changes in abundances of ctDNA mutations from UTUC-typical variant genes. The patient remained free of recurrence or metastasis as of this publishing, over 2 years after the initial surgical treatment.

**Conclusion:**

Immunochemotherapy may be a promising first-line option for advanced or metastatic UTUC selected with specific genomic or phenotypic signatures, and blood-based analyses incorporating ctDNA profiling provide precise longitudinal monitoring.

## Introduction

Upper tract urothelial carcinoma (UTUC) is an uncommon subtype of malignancies located at renal pelvis or ureter, accounting for 5%–10% of urothelial carcinoma (UC). Although both UTUC and urothelial bladder cancer (UBC) originate from urothelium, increasing evidence suggested that they have similar histologic appearances but distinct clinical features. In general, UTUC shows higher tumor grade, more advanced stage, and worse prognosis (1). UTUC and UBC share many genomic alterations and expression profiles but at varying frequencies, which contribute to the differences in biological behaviors and responses to treatment (2). Because of its rarity, the management of UTUC is basically based on studies in the histologically homologous UBC. However, because UTUC is more invasive and worse differentiated, intensive strategies should be developed regarding disease evaluation, treatment, and surveillance.

UTUCs are often asymptomatic or with mild symptoms at initial stage, and more than 50% of patients manifest as muscle-invasive or non–organ-confined disease at the time of diagnosis. High-risk non-metastatic UTUC should be treated with radical nephroureterectomy (RNU), and adjuvant platinum-based chemotherapy improves disease-free survivals for those with locally advanced disease, after complete lymphadetectomy (3). In the setting of metastatic or unresectable UTUC, platinum-based chemotherapy also becomes the first-line care, in view of benefits extrapolated from UBC. Patients with UTUC may present with impaired renal function after RNU and fail to tolerate the full-dose delivery of platinum drugs, especially cisplatin. In 2016, the first immune checkpoint inhibitor (ICI) for the systemic immunotherapy of patients with UC was approved, marking the beginning of the “immunotherapy revolution” with the publication of several noteworthy studies in the following years. The less toxic ICIs against programmed death-1 or its ligand (PD-1/PD-L1) are recommended for patients unfit for cisplatin or those refractory to first-line chemotherapy (4). Nonetheless, as alternative choices for cisplatin-based chemotherapy, pembrolizumab and atezolizumab first-line monotherapy only obtained objective response rate (ORR) of 28.6% and 22.9%, respectively, in phase 2 trials enrolling patients with cisplatin-ineligible UC (5, 6). Moreover, UBC-based strategies bring uncertainty to the minority individuals with UTUC, who seem to lack comparable responses to either chemotherapeutic or ICI agents in the real word (7, 8). First- or second-line trials on immunochemotherapy (ICT) have been carried out; however, overwhelming advantage over current chemo- or immuno-monotherapy has not yet been identified. In the era of next-generation sequencing, much more genomic or molecular information can be obtained from tumor and blood samples for comprehensive evaluation and personalized therapeutic regimens.

There have been a number of case studies regarding ICI-combined treatment in patients with UC; however, none has specially focused on ICT regimens for UTUC. Here, we present a case who received nephroureterectomy and first-line ICT for highly aggressive TP53/MDM2-mutated UTUC with ERBB2 mutations, luminal immune-infiltrated contexture, and non-mesenchymal state. The disease course was monitored by radiographic examinations, laboratory tests, and circulating tumor DNA (ctDNA) analyses, all of which demonstrated complete remission over two years after diagnosis.

## Case presentation

**Figure 1:**
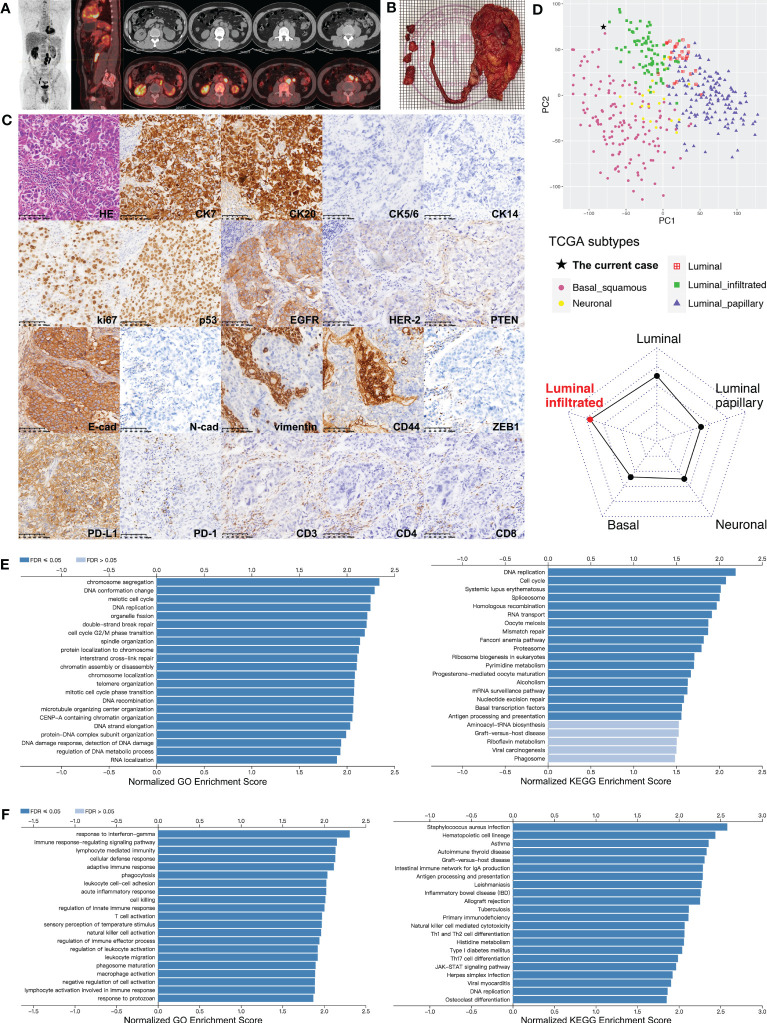
Comprehensive analyses of the UTUC lesions from the patient. **(A)** PET-CT scan performed before surgery for the patient indicating malignant lesions involving left upper-middle ureter and surrounding lymph nodes attaching aorta and psoas major. **(B)** Surgical specimens of left kidney, ureter, and resected lymph nodes. **(C)** Pathologic analysis for the primary tumor and staining of markers for tumor subtype, proliferation, EMT, and immune status (in order from top to bottom row). Strongly positive: CK7, CK20, ki-67, p53, EGFR/HER-1, E-cadherin, and PD-L1; mildly positive: HER-2; negative: CK5/6, CK14, PTEN, CD44, N-cadherin, vimentin, and ZEB1; infiltrating lymphocytes: 15% PD-1^+^, 50% CD3^+^, 40% CD4^+^, and 10% CD8^+^. **(D)** Transcriptome subtype classification for the patient’s primary tumor using TCGA classifier based on RNA sequencing data. Left panel: principal component analysis to visualize separation among subtypes (luminal, luminal-papillary, luminal-infiltrated, basal-squamous, and neuronal) of TCGA UC cohort, and this patient clustered with the luminal-infiltrated subset; right panel: cosine similarity measure classifying the current case into the luminal-infiltrated subtype. **(E)** Bar charts for GO (left) and KEGG (right) categories significantly enriched in TP53-mutant cases of TCGA-UC dataset. **(F)** Bar charts for GO (left) and KEGG (right) categories significantly enriched in ERBB2-mutant cases of TCGA-UC dataset. CK, cytokeratin; EGFR/HER, human epidermal growth factor receptor; FDR, false discovery rate; GO, Gene Ontology; HE, hematoxylin-eosin; KEGG, Kyoto Encyclopedia of Genes and Genomes; PD-1, programmed death-1; PD-L1, programmed death-1 ligand; PTEN, phosphatase and tensin homolog; TCGA, The Cancer Genome Atlas; ZEB1, zinc finger E-box-binding homeobox-1.

In October 2020, a 50-year-old man complaining of left flank pain was referred to our center after left hydronephrosis was found by ultrasonography in another institution. The patient had no history of smoking or exposure to carcinogenic chemicals. Computed tomography (CT) urography showed left hydronephrosis, ipsilateral upper-middle ureter thickening, and locally enlarged lymph nodes. Elevations in routinely detected serum creatinine, carbohydrate antigen 72-4 (CA72-4), and cytokeratin-19 (CK19) fragments (CYFRA21-1) were found. Patient received ^18^F-fluorodeoxyglucose positron emission tomography (PET)/CT scan, which confirmed malignancy involving 4.5-cm segment of left proximal ureter. Surrounding hypermetabolic lymph nodes extended to paraaortic region, and no distant metastasis was found (**Figure 1A**). Plasma was collected for ctDNA sequencing and mutations found in tumor suppressor TP53, oncogene ERBB2, and chromatin-remodeling genes lysine(K) demethylase 6A (KDM6A) and lysine(K) methyltransferase 2D (KMT2D) (**Table 1**). Retroperitoneoscopic RNU, regional lymphadenectomy, and cystoscopy were planned and performed, whereas a few paraaortic metastatic lesions were not successfully removed because of deep location and surrounding adhesion (**Figure 1B**). The pathology showed ureteral high-grade UC with periureteral lymphatic metastases (pT_3_N_2_M_0_), and surgical margins were all negative. Immunohistochemical (IHC) staining was performed for representative markers of subtypes (cytokeratins), tumor growth (i.e., ki-67 and p53), epithelial–mesenchymal transition (EMT) (i.e., cadherins and vimentin) and immune status (PD-L1 and lymphocyte markers) (**Figure 1C**). It was characterized as proliferative, luminal-like, and non-mesenchymal tumor with inflamed microenvironment. Tumor tissue was also subjected to DNA exome and RNA transcriptional sequencing. Main somatic gene alterations involved missense mutations of TP53 and ERBB2 as well as truncating mutations of KDM6A, KMT2D, AT-rich interactive domain-containing protein 1A (ARID1A), and mismatch repair gene MLH1 (**Supplementary Tables S1, S2**), and tumor mutation burden (TMB) was 6.3 mutations per megabase (muts/Mb). According to the UTUC mutational classifications, this case belongs to TP53/MDM2-mutated subtype with probably worst prognosis (9).

**Table 1:** Changes in ctDNA mutations and corresponding abundances of TP53, KDM6A, KMT2D, ARID1A, and ERBB2 during the disease course of the patient.

Mutation	Abundances of variants			
	Baseline (month 0)	Pre-ICT (month 1.5)	Post-ICT (month 9)	Post-ICT (month 13)
TP53 c.853C>G (p.E285K)	1.60%	5.4%	N.D.	N.D.
TP53 c.184G>A (p.E62K)	1.20%	4.6%	N.D.	N.D.
TP53 c.861C>T (p.E287D)	N.D.	5.4%	N.D.	N.D.
KDM6A c.3790C>T (p.Q1264X)	2.00%	4.90%	N.D.	N.D.
KMT2D c.11470C>T (p.H3824Y)	1.10%	2.20%	N.D.	N.D.
KMT2D c.12524C>G (p.P4175R)	0.92%	3.00%	N.D.	N.D.
KMT2D c.11097C>A (p.F3699L)	0.62%	2.90%	N.D.	N.D.
KMT2D c.12850C>T (p.Q4284X)	N.D.	2.90%	N.D.	N.D.
ERBB2 c.2327G>T (p.G776V)	0.79%	5.20%	N.D.	N.D.
ERBB2 c.929C>T (p.S310F)	N.D.	1.30%	N.D.	N.D.
ERBB2 c.314C>T (p.T105I)	N.D.	1.20%	N.D.	N.D.
ARID1A c.261_278del (p.A88_G93del)	N.D.	N.D.	0.57%	N.D.

N.D., not detected.

Furthermore, the UC subset of The Cancer Genome Atlas (TCGA) database was employed to deeply understand the features of this patient. Using TCGA classifier for UC (10), his lesion was clustered with luminal-infiltrated subtype based on RNA sequencing data (**Figure 1D**). We also performed gene set enrichment analysis (GSEA) for TCGA-UC dataset to process gene expression profiles (GEPs) related with key genomic mutations identified in the current case. Gene Ontology (GO) and Kyoto Encyclopedia of Genes and Genomes (KEGG) analyses suggested that highly expressed genes in TP53-mutant tumors were significantly associated with biological processes and pathways regarding assembly and repair of DNA or chromatin, i.e., DNA replication, DNA damage response, and mismatch repair (**Figure 1E**; **Supplementary Figure S1**). Among ERBB2-mutant UC cases, GSEA showed that immune-activating categories [i.e., response to Interferon gamma (INF-γ), natural killer (NK)–cell activation, and T helper type 1 (Th1)- and T helper type 2 (Th2)-cell differentiation] were highly enriched (**Figure 1F**; **Supplementary Figure S2**).

**Figure 2:**
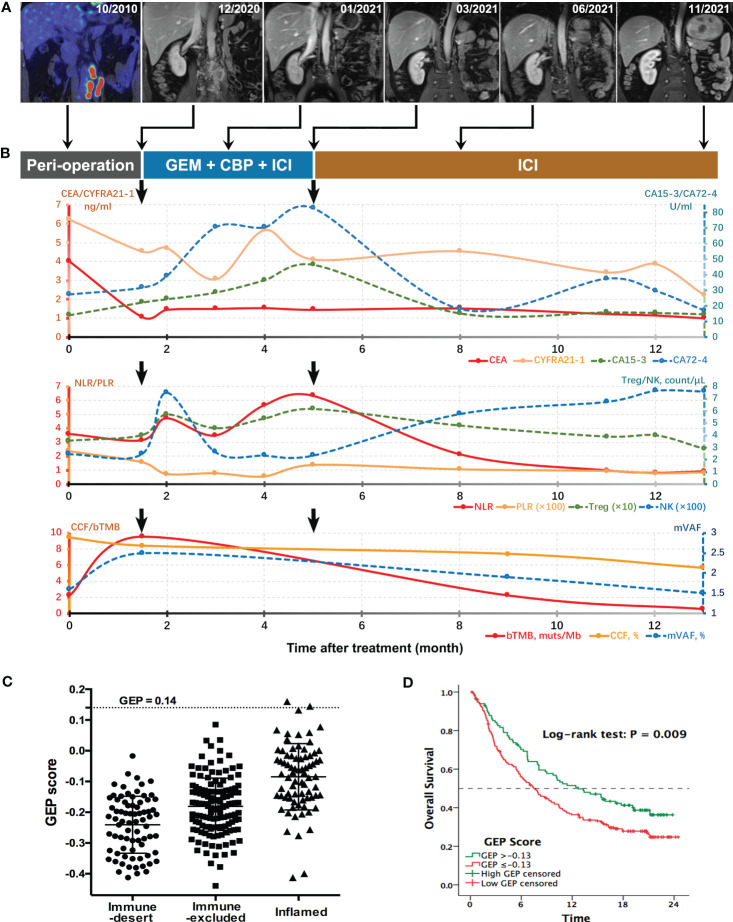
Longitudinal surveillance of the patient’s treatment course. **(A)** Preoperative PET-CT and serial MRI scans for monitoring the treatment response of lymphatic metastases at different follow-up time points. **(B)** Dynamic changes of serum tumor markers, systemic inflammatory indices and ctDNA parameters during the treatment. Postoperative (month 1.5) levels of CEA, CYFRA21-1, and CCF were decreased as compared with baseline values corresponding with surgical resection of primary tumor and most of the lymphatic metastases, whereas increased levels of CA72-4, CA15-3, bTMB, and mVAF were supported by rapid progression of residual metastatic lymph nodes as indicated by MRI scans. Durable response to ICT was supported by constantly ascending NK-cell count and descending trends of all other indices, especially Treg count, bTMB, and mVAF. **(C)** GEP scores of tumors with different immune status in IMvigor210 cohort with metastatic UC. **(D)** Overall survival curves for high-GEP and low-GEP subgroups of the anti–PD-1–treated IMvigor210 cohort. bTMB, blood tumor mutation burden; CA, carbohydrate antigen; CBP, carboplatin; CCF, cancer cell fraction; CEA, carcinogenic embryonic antigen; CYFRA21-1, cytokeratin-19 fragments; GEM, gemcitabine; GEP, T-cell–inflamed gene expression profile; ICI, immune checkpoint inhibitor; mVAF, mean variant allele frequency; NK, natural killer; NLR, neutrophil-to-lymphocyte ratio; PLR, platelet-to-lymphocyte ratio; Treg, regulatory T cell.

Postoperatively, four times of weekly intravesical gemcitabine instillation were administered. However, patient complained about gradually severe left flank pain simultaneously. Magnetic resonance imaging (MRI) showed a significant enlargement of residual lymphatic metastases at six weeks after surgery (**Figure 2A**). Compared with preoperative values, higher levels of serum tumor biomarkers CA72-4, CA15-3, and regulatory T (Treg)–cell count were observed. The ctDNA test revealed elevated abundances of mutations from TP53, ERBB2, KDM6A, and KMT2D (**Table 1**). Further ctDNA analyses showed a decrease in the cancer cell fraction (CCF) but remarkable increases in blood TMB (bTMB) and mean variant allele frequency (mVAF), which was calculated as the mean of VAFs of somatic mutated genes for each specimen (**Figure 2B**). All above findings suggested disease progression postoperatively. Meanwhile, RNA sequencing data of tumor tissue were analyzed to retrieve T-cell–inflamed GEP, a pan-cancer immunotherapy response biomarker (11). We validated GEP in an ICI-treated IMvigor210 cohort with metastatic UC, among whom the score (range, −0.44~0.16) successfully distinguished tumors with different immune status, as demonstrated by the average increasing in sequence from immune desert, through immune-excluded, to inflamed subgroup (−0.24 vs. −0.18 vs. −0.08, P < 0.001). IMvigor210 cohort was divided into high-GEP (one of the three) and low-GEP (two of the three) subgroups using the score cutoff of −0.13. High-GEP subjects had significantly longer overall survivals than low-GEP counterparts by a median of 5.1 months (P = 0.009), indicating the usefulness of GEP score in prediction of response to ICI agents. GEP score of this patient was high at 0.14, predicting active immunity and favorable response to PD-1 blockage (**Figures 2C, D**).

On the basis of the aforementioned analyses, ICT treatment was started in December 2020, consisting of off-label PD-1 inhibitor sintilimab [200 mg, day 1 (D1)], gemcitabine (1,000 mg/m^2^, D2/9), and carboplatin (area under the curve of 5–6, D3), every 3 weeks. Sintilimab (Innovent Biologics, Suzhou) is the first approved PD-1 antibody in China with affordable price. The patient tolerated the combined treatment well, and myelosuppression was dealt with granulocyte colony-stimulating factor (G-CSF) or thrombopoietin, if indicated. MRI showed shrinks of lymphatic metastases at cycle 3 and most regressed at cycle 6. Flank pain was gradually relieved, although CA72-4, CA15-3, and neutrophil-to-lymphocyte ratio (NLR) were on the rise. Since cycle 7, sintilimab monotherapy instead of ICT was continued. NK-cell count went steadily up afterward, and CA72-4, CA15-3, and NLR descended significantly to liminal levels. Carcinoma embryonic antigen (CEA), CYFRA21-1, Treg-cell count, and platelet-to-lymphocyte ratio (PLR) also showed declining trends. MRI demonstrated complete remission of lymphatic metastases at cycle 16. Meanwhile, ctDNA analyses confirmed sustained decreases in CCF, bTMB, and mVAF (**Figure 2**). Specifically, none of previously detected variants was present at the last detection (**Table 1**). Sintilimab was maintained up to 1 year. As of this publishing, the patient remained free of recurrence or metastasis, as shown by radiographic examinations and cystoscopy in March 2023, 16 months after the end of sintilimab treatment.

## Discussion

To date, UTUC and UBC are still generally regarded as a homologous entity in both clinical trials and practices, among which their prognostic discrepancies remain neglected. UTUC is usually not diagnosed until obvious symptoms provide clues at relatively late stage and the oncologic outcomes of invasive UTUC are frequently unfavorable despite surgical treatment. For locally advanced or metastatic UC, UTUC lesions show worse oncologic outcomes than pathologically matched lower tract counterparts (1). Although ORR of first-line platinum–based chemotherapy exceeds 45% in metastatic UBC, it may reach as low as 28.7% for UTUC (7). It is crucial to identify chemo-sensitive cases in UC cohorts, especially for UTUC. EMT process may mediate resistance to platinum (12). Missense activating mutations of ERBB2 that support cellular transformation, like S301F variant, exclusively exist in platinum responders of UC (13). Another ERBB2 mutation G776V has not been characterized in UC, yet it is an activating mutation within the kinase domain (14). Despite lacking reliable chemo-response biomarkers in UC, above mutations and non-mesenchymal state may predict favorable response to carboplatin for this patient. Nonetheless, rapid progression of residual nodal metastases demonstrates his lesions of extraordinary malignancy. Fujii et al. (9) profiled the genomic landscape of UTUC and identified five discrete mutational classifications, namely, hyper-mutated, TP53/MDM2-mutated, RAS-mutated, FGFR3-mutated, and triple-negative subtypes. This case could be confirmed as TP53/MDM2-mutated subtype, which would emerge as aggressive clinical course (9). The tumor also combines classic luminal IHC-phenotype of CK5/6^−^CK20^+^CD44^−^ and loss of PTEN expression, both of which correlate with worse outcomes in UTUC (15, 16). For such highly aggressive case, there is a concern that chemotherapy has reached a plateau in terms of efficacy. Personalized regimens based on comprehensive evaluation are needed.

ICI agents have become standard options for platinum-resistant UC, but their roles are still not robust in treat-naïve cases. **Table 2** summarizes first-line ICI trials in cohorts with advanced or metastatic UC incorporating patients with UTUC. ORRs of ICI monotherapies ranged around merely 20%–30%, nowhere near chemotherapeutic responses. Currently, only pembrolizumab and atezolizumab were approved for cisplatin-ineligible PD-L1^+^ cases based on impressive ORRs for high PD-L1 expressors in KEYNOTE-052 and IMvigor210, among which subgroup analyses of UTUC versus UBC showed numerically inferior and superior results, respectively (5, 6). To optimize first-line regimens, subsequent studies have been focusing on addition of ICIs to chemotherapy. IMvigor130 results showed near-doubled complete responses and significantly prolonged progression-free survival from synchronous combination plus further maintenance of atezolizumab relative to chemotherapy alone (17), but, in KEYNOTE-361, the concurrent pembrolizumab combination failed to render overwhelming advantages (18). Using another PD-L1 inhibitor avelumab, phase 3 trial JAVELIN Bladder 100 demonstrated additional ICI maintenance significantly prolonged overall survival, as compared with best supportive care, among patients with UC who had well-controlled disease after first-line chemotherapy (19). Similar sequential integration of platinum-based chemotherapy and pembrolizumab also showed the superiority in phase 2 trial NCT02500121 (20). However, when avelumab was administered prior to chemotherapy in INDUCOMAIN, the induction combination approach brought about increased risks of progression than chemotherapy alone (21). Among these trials, as compared with UBC subsets, patients with UTUC obtained comparable benefits from atezolizumab combination in IMvigor130, whereas less favorable responses to avelumab maintenance in JAVELIN 100 (17, 19).

**Table 2:** Overview of first-line clinical trials on immune checkpoint inhibitors alone or combined with chemotherapy for advanced or metastatic urothelial carcinoma.

Drugs Studies (phase)	Interventions	Sample size UTUC/LTUC	ORR		Median OS [months (95% CI)]		
			Overall	UTUC/LTUC	Overall	UTUC/LTUC	
Pembrolizumab							
KEYNOTE-052 (II)	Pembrolizumab	69/300	28.6%	26.1%/29.3%	11.3 (9.7–13.1)	10.8 (7.6–17.0)/11.5 (9.7–13.1)	
KEYNOTE-361 (III)	A: Pembrolizumab; B: GCis/GCpb; C: Combined	211/799	A: 30.3%; B: 44.9%; C: 54.7%	N.A.	B: 14.3 (12.3–16·7); C: 17.0 (14.5–19.5)	N.A.	
NCT02500121 (II)	A: Platinum followed by pembrolizumab; B: Platinum followed by placebo	N.A. (108 in total)	A: 23%; B: 10% (after chemotherapy, crossover allowed)	N.A.	A: 22.0 (12.9–NE); B: 18.7 (11.4–NE)	N.A.	
ABLE (II)	Pembrolizumab + abraxane	11/25	50.0%	N.A.	Pending	
Atezolizumab							
IMvigor210 (II)	Atezolizumab	33/85	22.9%	39.4%/16.5%	15.9 (10.4-NE)	NE (15·3–NE)/13·4 (6·7–NE)	
IMvigor130 (III)	A: Atezolizumab; B: GCis/GCbp; C: Combined	312/901	A: 22.8%; B: 43.8%; C: 47.4%	B: 40.0%/44.6%; C: 49.6%/46.9%	B: 13.4 (12.0–15.2); C: 16.0 (13.9–18.9)	B: 13.5 (10.1–17.6)/13.4 (11.7–15.3); C: 16.9 (12.5–25.5)/15.8 (12.9–18.9)	
NCT03093922 (II)	GCis before or after atezolizumab; estimated 32 enrolled					
Avelumab							
ARIES (II)	Avelumab (for PD-L1^+^ve, cisplatin-unfit patients )	N.A. (71 in total)	22.5%	N.A.	10.0 (5.7–14.3)	N.A.	
JAVELIN Bladder 100 (III)	A: GCis/GCbp followed by avelumab; B: GCis/GCbp followed by placebo	187/513	A: 9.7%; B: 1.4% (after chemotherapy)	N.A.	A: 21.4 (18.9–26.1); B: 14.3 (12.9–17·9)	A:19.9 (15.3–NE)/22.5 (19.0–28.3); B:17.4 (12.8–33.0)/14.1 (11.8–17.9)	
INDUCOMAIN (II)	A: GCbp chemotherapy ; B: GCbp + avelumab (induction + maintenance)	18/67	A: 53.5%; B: 57.1%	N.A.	A: 13.2 (12.5–18.4); B: 10.5 (6.9–NE)	N.A.	
GCISAVE (II)	Two arms: GCis and GCis + avelumab; estimated 90 enrolled					
Nivolumab, ipilimumab							
CheckMate901 (III)	Three arms: Nivolumab + ipilimumab, nivolumab + GCis, and GCis/GCbp; estimated 1,307 enrolled					
Durvalumab, tremelimumab							
DANUBE (III)	A: Durvalumab; B: Durvalumab + tremelimumab; C: GCis/GCbp	221/810	A: 25.7%; B: 36.3%; C: 49.1%	N.A.	A: 13.2 (10.3–15.0); B: 15.1 (13.1–18.0); C: 12.1 (10.9–14.0)	B: 15.1/15.1; C: 10.9/12.7	
NILE (III)	Three arms: Durvalumab + GCis/GCbp, durvalumab + tremelimumab + GCis/GCbp, and GCis/GCbp; estimated 1,292 enrolled.					

UTUC, upper tract urothelial carcinoma; LTUC, lower tract urothelial carcinoma; ORR, objective response rate; OS, overall survival; CI, confidential interval; NE, non-estimable; GCis, gemcitabine plus cisplatin; GCbp, gemcitabine plus carboplatin.

Aforementioned confusing outcomes of ICIs might be attributed to uncertain association of PD-L1 status with treatment response and lack of sub-selection by molecular subtypes. In fact, the mainstream features of UTUC may make a dent in immunotherapeutic response. Mean TMB levels of UTUC are lower than those of UBC (2.3–2.9 vs. 5.8 muts/Mb), and most sporadic UTUCs have immunotherapy-unfriendly luminal-papillary T-cell–depleted landscape (9, 22). For this patient, antitumor immune response was preliminarily forecasted by high PD-L1 expression and TMB level. The case also shared the characteristics of TCGA luminal-infiltrated (cluster II) tumors with lymphocyte infiltration, which could be ICI-sensitive signatures (6, 10, 23). Moreover, he harbored ERBB2 mutations, potentially useful predictors of responders to ICI treatment in gallbladder cancer (24). Our TCGA-based analysis also shows that ERBB2-mutated UC tumors are likely to manifest with immune activation, suggesting potentially favorable immunotherapeutic response. Moreover, the mesenchymal state, which frequently occurs in luminal-infiltrated UC but attenuates ICI response by hindering lymphocytes from infiltrating tumor parenchyma (10, 25), was not identified in the current case. On the basis of the above evidence, he received combined ICI treatment and obtained sustained response for more than 2 years. Although chemotherapy renders initial responses, balance of immune cells may be broken. NK cells would expand in responders of immunotherapy but decline after chemotherapy (26, 27). G-CSF administration during chemotherapy induces immune tolerance by upregulating immunosuppressive Treg cells, the abundance of which may predict for poor clinical outcomes in many cancer types (28–30). Sintilimab combination here may counteract chemotherapy-induced immunocompromise and exert durable antitumor activity, as indicated by count-time curves of circulating NK and Treg cells.

The initial stage of UTUC surveillance usually depends on imaging techniques, which are insensitive if target lesion shrinks. Serum tumor and inflammatory markers are widely or routinely used but rough, lagging or even invalid for urologic malignancies. For example, sustained response to the latter sintilimab monotherapy here was accompanied by decreasing trends of previously elevated pan-cancer tumor makers CA15-3 and CA72-4, as well as CYFRA21-1, which may be specific for UTUC (31). Nevertheless, fluctuation of CYFRA21-1 and elevations of CA72-4 and CA15-3 during the initial six ICT cycles for the patient were opposite to the gradually eradicated lesions as shown by MRI scans, probably attributed to the releases of these antigens from damaged lesions into bloodstream. Levels of NLR and PLR were also influenced by administration of chemotherapeutic agents and marrow stimulants during this period. By contrast, the ctDNA profiling is a novel promising method of liquid biopsy. Neither affected by non-tumor factors nor subjected to tumor heterogeneity, ctDNA contains comprehensive information about tumor genomes which would not be acquired from conventional plasma-based analyses. Here, downtrends of CCF, bTMB, and mVAF agreed with sustained response to ICT. However, during initial perioperative stage, the decline in CCF was in accord with surgical reduction of tumor burden rather than enhanced malignancy of residual lesions, which were accurately mirrored by rises in bTMB and mVAF. Both bTMB dynamics and mVAF kinetics are concordant with tumor activity and therapeutic response, even for radiologically stable disease (32, 33). The ctDNA-based mVAF and bTMB represent abundance and amount of mutant genes from tumor cells, respectively, among which specific pathogenic variants are associated with progression and invasiveness of disease. Dynamic changes in mutation abundances of TP53, ERBB2, and KDM6A, which frequently mutate in UC, are inherent embodiments of the disease course in this patient. The elimination of genomic variants in the last ctDNA analysis is more convincing than negative imaging finding when determining complete response. Further studies are needed to confirm specific variants as surrogate biomarkers in longitudinal surveillances of UTUC cohorts. It also calls for minimal residual disease detection, which demands ultra-sensitive ctDNA testing. Recent advances in molecular and computational biology have improved the limit of detection for ctDNA. For example, genome-wide mutational integration technique would overcome the limitation of ctDNA abundance and empower treatment optimization in low–disease-burden oncology care (34). DNA methylation or other epigenetic features are also complementary to somatic mutation tracking by reflecting chromatin state of tumor cells. Incorporating epigenomics-based analysis would improve sensitivity compared with conventional tumor-naïve somatic gene alterations alone (35, 36).

Notably, the major limitation of this study is that only one case was reported. Further research studies will be carried out to validate the association of genomic and phenotypic signatures with response to ICT among more patients with UTUC. Because UTUC-specific data are still relatively limited, clinicians following UBC evidence–based treatment guidelines are often left to their own devices to tackle heterogeneity in treatment efficacy. Personalized trial designs, such as n-of-1 trials or single-case studies, would address this fundamental problem to some extent and suggest a road map of action priorities to accomplish precision medicine’s vision for identifying the best treatments for each patient. The current real-world UTUC case has been successfully treated on the basis of the personalized, precise, and data-driven exploration, and we point out that this experience may provide some hints on optimal management of UTUC.

In summary, this study demonstrated the promising effect of first-line ICT for highly aggressive TP53/MDM2-mutated advanced or metastatic UTUC with features more than PD-L1 positivity, including ERBB2 mutations, luminal immune-infiltrated contexture, and non-mesenchymal state. As supplementary of radiographic examinations, ctDNA profiling provides precise longitudinal monitoring for treatment response. Prospective studies are needed to assess outcomes of ICT regimens versus current first-line chemo- or immune-monotherapy in selected patients with UTUC, among whom genomic or phenotypic signatures may be determinants of efficacies.

## Data availability statement

The original contributions presented in the study are included in the article/**Supplementary Material**. Further inquiries can be directed to the corresponding author.

## Ethics statement

Written informed consent was obtained from the individual(s) for the publication of any potentially identifiable images or data included in this article.

## Author contributions

BY, XY and TX developed the project. JC, BP and TX were involved in surgical treatment of the patient. BY, TX and HG were in charge of immunochemotherapy for the patient. JX and YH collected the data. TX and YH analyzed the data. TX and HG wrote the main manuscript. JX prepared tables. YH prepared figures. All authors contributed to the article and approved the submitted version.
