# Supplementary material for: Sustained complete response to first-line immunochemotherapy for highly aggressive TP53/MDM2-mutated upper tract urothelial carcinoma with ERBB2 mutations, luminal immune-infiltrated contexture, and non-mesenchymal state: a case report and literature review
Source: Front Oncol. 2023 Jun 23;13:1119343. doi: 10.3389/fonc.2023.1119343 (PMC10328386; doi:10.3389/fonc.2023.1119343)
Supplement: Supplementary file 1 [file DataSheet_1.pdf]

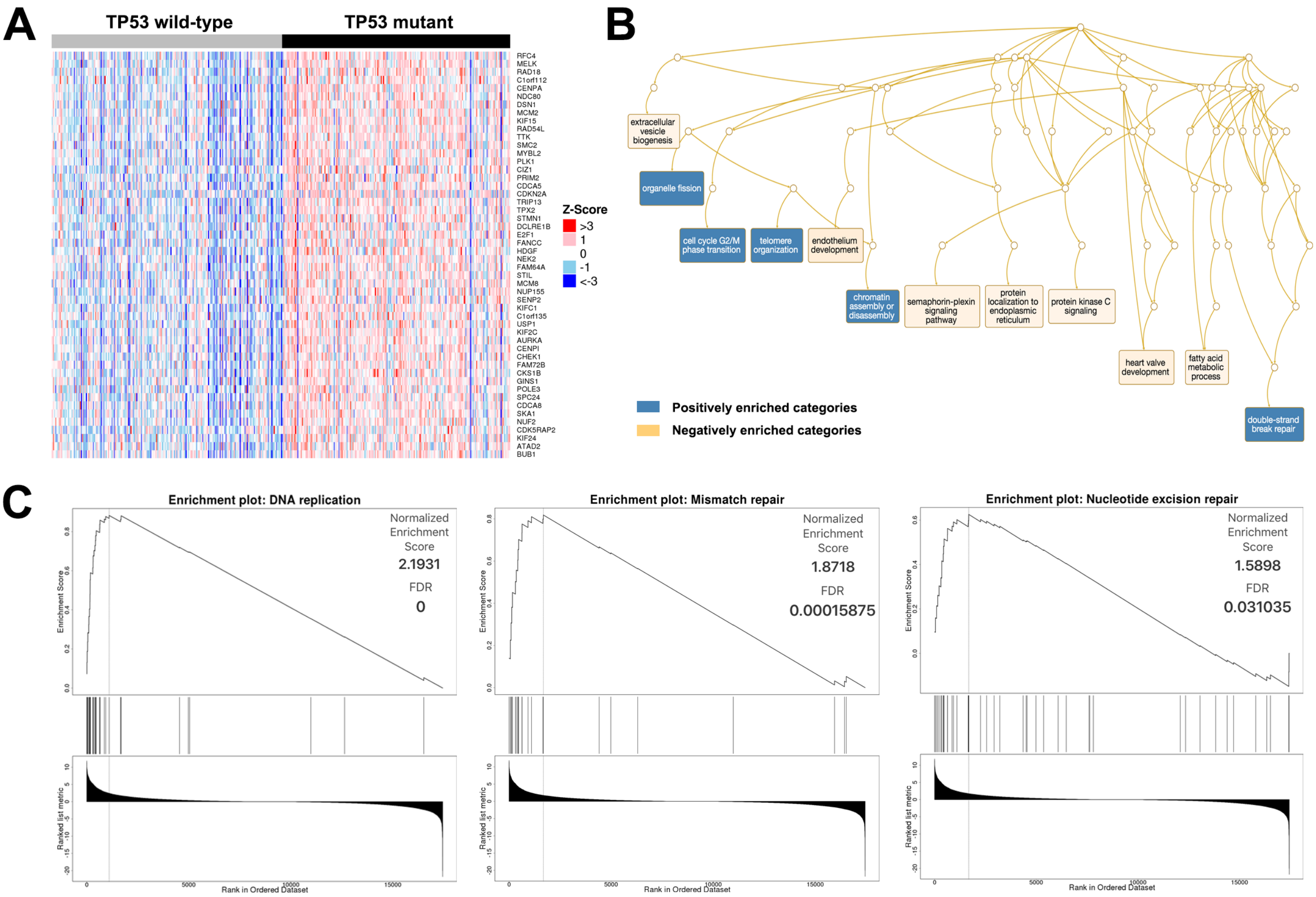

Figure S1 Gene set enrichment analyses of differential expressed genes correlated with TP53 mutations in TCGA-UC dataset. **(A)** Heatmap of top positively enriched genes correlated with TP53 mutations. **(B)** Directed acyclic graph of positively and negatively enriched GO categories correlated with TP53 mutations. **(C)** Representative enriched KEGG categories positively correlated with TP53 mutations.

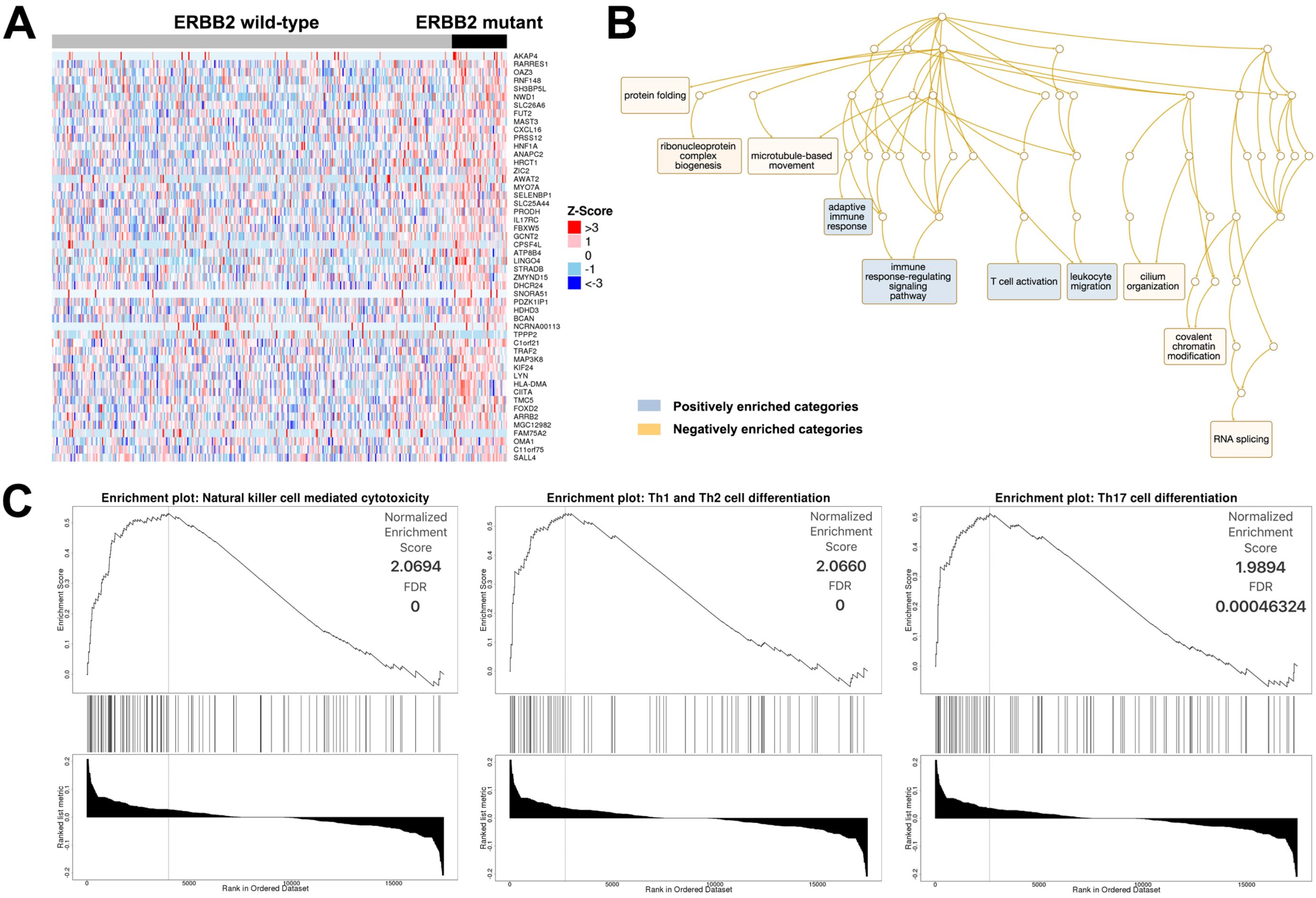

Figure S2 Gene set enrichment analyses of differential expressed genes correlated with ERBB2 mutations in TCGA-UC dataset. **(A)** Heatmap of top positively enriched genes correlated with ERBB2 mutations. **(B)** Directed acyclic graph of positively and negatively enriched GO categories correlated with ERBB2 mutations. **(C)** Representative enriched KEGG categories positively correlated with ERBB2 mutations.

Table S1 Gene list of DNA exome sequencing (642-gene panel) for the patient

ABL1	ACO1	ACVR1	ACVR1B	ACVR2A	ACVR2B	ADNP	AFF3
AJUBA	AKT1	AKT2	AKT3	ALK	ALKBH6	ALOX12B	ALPK2
AMER1	AMFR	ANK3	ANKRD11	APC	APLNR	APOL2	AR
ARAF	ARHGAP35	ARID1A	ARID1B	ARID2	ARID5B	ASXL1	ASXL2
ATM	ATP5B	ATR	ATRX	AURKA	AURKB	AXIN1	AXIN2
AXL	AZGP1	B2M	B4GALT3	BAP1	BARD1	BBC3	BCL10
BCL2	BCL2L1	BCL2L11	BCL6	BCLAF1	BCOR	BCORL1	BHMT2
BICC1	BIRC3	BLM	BMPR1A	BRAF	BRCA1	BRCA2	BRD4
BRE	BRIP1	BTK	C3orf70	CACNA1D	CALR	CAP2	CARD11
CASP8	CBFB	CBL	CCDC120	CCDC6	CCND1	CCND2	CCND3
CCNE1	CDC27	CDKN1B	CHD4	COL5A3	CTLA4	DAXX	DNAH12
E2F3	EIF4E	EPHA7	ERCC2	ETV6	FAM175A	FANCG	FGF3
FIP1L1	FOXA2	GAPDH	GNAQ	GSK3B	HIST1H1C	HIST1H3F	HLA-A
ICOSLG	IGF2	INHBA	IRF1	JAK2	KEAP1	KMT2D	CD1D
CDC73	CDKN1C	CHD8	CREBBP	CTNNB1	DCUN1D1	DNAJB1	EED
ELF3	EPHB1	ERCC3	EWSR1	FAM46C	FANCI	FGF4	FLCN
FOXL2	GATA1	GNAS	GUSB	HIST1H1E	HIST1H3G	HLA-B	ID3
IKBKE	INPP4A	IRF4	JAK3	KEL	KRAS	CD274	CDH1
CDKN2A	CHEK1	CRIPAK	CUL3	DDR2	DNER	EGFL7	EML4
EPHB6	ERCC4	EXT1	FANCA	FANCL	FGFBP1	FLG	FOXO1
GATA2	GNB1	H3F3A	HIST1H2BD	HIST1H3H	HLA-C	IDH1	IKZF1
INPP4B	IRF6	JUN	KIF5B	LATS1	CD276	CDK12	CDKN2B
CHEK2	CRKL	CUL4B	DDX3X	DNMT1	EGFR	EP300	EPPK1
ERCC5	EXT2	FANCB	FANCM	FGFR1	FLI1	FOXP1	GATA3
GNPTAB	H3F3B	HIST1H3A	HIST1H3I	HNF1A	IDH2	IL10	INPPL1
IRS1	KMT2B	KIT	LATS2	CD70	CDK4	CDKN2C	CIC
CRLF2	CUX1	DDX5	DNMT3A	EGR3	EPCAM	ERBB2	ERG
EZH1	FANCC	FAT1	FGFR2	FLT1	FOXQ1	GEN1	GOT1
H3F3C	HIST1H3B	HIST1H3J	HOXB13	IFNGR1	IL6ST	INSR	IRS2
KDM5A	KLF4	LCTL	CD74	CDK6	CEBPA	CNBD1	CSF1R
CXCR4	DIAPH1	DNMT3B	EIF1AX	EPHA2	ERBB3	ERRFI1	EZH2
FANCD2	FAT4	FGFR3	FLT3	FRMD7	GLI1	GPS2	HDAC1
HIST1H3C	HIST1H4E	HRAS	IFNGR2	IL7R	INSRR	ITGB7	KDM5C
KLHL8	LIFR	CD79A	CDK8	CENPA	CNKSR1	CSF3R	CYLD
DICER1	DOT1L	EIF2S2	EPHA3	ERBB4	ESR1	EZR	FANCE
FBXW7	FGFR4	FLT4	FUBP1	GNA11	GREM1	HDAC2	HIST1H3D
HIST2H3D	HSD3B1	IGF1	ING1	INTS12	ITPKB	KDM6A	KMT2A
LMO1	CD79B	CDKN1A	CEP76	COL5A1	CTCF	CYP17A1	DIS3
DROSHA	EIF4A2	EPHA5	ERCC1	ETV1	FAM166A	FANCF	FGF19
FH	FOXA1	FYN	GNA13	GRIN2A	HGF	HIST1H3E	HIST3H3

HSP90AB1	IGF1R	INHA	IPO7	JAK1	KDR	KMT2C	LRP1B
LRRK2	MALT1	MAP2K1	MAP2K2	MAP2K4	MAP3K1	MAP3K13	MAP4K3
MAPK1	MAPK3	MAPK8IP1	MAX	MBD1	MCL1	MDC1	MDM2
MDM4	MECOM	MED12	MED23	MEF2B	MEN1	MET	MGA
MICALCL	MITF	MLH1	MLH3	MORC4	MPL	MPO	MRE11A
MSH2	MSH6	MST1	MST1R	MTOR	MUC17	MUTYH	MXRA5
MYB	MYC	MYCL	MYCN	MYD88	MYOCD	MYOD1	NAB2
NAV3	NBN	NBPF1	NCOA3	NCOR1	NCOR2	NEGR1	NF1
NF2	NFE2L2	NFE2L3	NFKBIA	NKX2-1	NKX3-1	NOTCH1	NOTCH2
NOTCH3	NOTCH4	NPM1	NRAS	NSD1	NTN4	NTRK1	NTRK2
NTRK3	PAK1	PCBP1	PDSS2	PIK3CD	PMAIP1	POU2F2	PRKAR1A
PTPRK	RAD17	RAD54L	REL	RNF43	RPS6KB2	SDHA	SETBP1
SIN3A	SMAD2	SMO	SPEN	STAT6	TACC3	TCF7L2	TGFBR2
TOP1	TRIM23	TXNDC8	WASF3	XRCC1	ZBTB16	ZRANB3	NUP210L
PAK7	PCNA	PGR	PIK3CG	PMS1	PPM1D	PRKDC	PTPRS
RAD21	RAD9A	RET	ROS1	RPTOR	SDHAF2	SETD2	SIRT4
SMAD3	SND1	SPOP	STK11	TAF1	TCP11L2	TIMM17A	TP53
TRRAP	TYK2	WRN	XRCC2	ZFHX3	ZRSR2	NUP93	PALB2
PDAP1	PHF6	PIK3R1	PMS2	PPP2R1A	PRX	PTPRT	RAD50
RAF1	RFC1	RPL22	RRM1	SDHB	SETDB1	SIX1	SMAD4
SNX25	SRC	STK19	TAP1	TDRD10	TLR4	TP53BP1	TSC1
U2AF1	WT1	XRCC3	ZNF180	ODAM	PAPD5	PDCD1	PHOX2B
PIK3R2	PNRC1	PPP2R2A	PTCH1	QKI	RAD51	RARA	RFWD2
RPL5	RSBN1L	SDHC	SF3B1	SLC1A3	SMARCA4	SOCS1	SRSF2
STK40	TBC1D12	TERT	TMEM127	TP63	TSC2	USP9X	XIAP
XRCC4	ZNF471	OMA1	PARK2	PDCD2L	PIK3C2G	PIK3R3	POLD1
PPP6C	PTEN	RAB35	RAD51B	RASA1	RHEB	RPP30	RUNX1
SDHD	SGK1	SLC26A3	SMARCB1	SOS1	STAG2	STX2	TBL1XR1
TET1	TMPRSS2	TPX2	TSHR	VEGFA	XIRP2	XRCC5	ZNF483
OR4A16	PARP1	PDGFRA	PIK3C3	PIM1	POLE	PRDM1	PTPN11
RAB40A	RAD51C	RB1	RHOA	RPS15	RXRA	SELP	SH2B3
SLC44A3	SMARCD1	SOX17	STAT3	SUFU	TBX3	TET2	TNF
TRAF2	TSHZ2	VEZF1	XPA	XRCC6	ZNF521	OR52N1	PAX5
PDGFRB	PIK3CA	PLCG2	POLQ	PREX2	PTPRB	RAC1	RAD51D
RBM10	RICTOR	RPS2	RYBP	SEPT12	SH2D1A	SLC4A5	SMC1A
SOX2	STAT5A	SUZ12	TCEB1	TFE3	TNFAIP3	TRAF3	TSHZ3
VHL	XPC	YAP1	ZNF620	OTUD7A	PBRM1	PDPK1	PIK3CB
PLK2	POU2AF1	PRKACA	PTPRD	RAD1	RAD52	RECQL4	RIT1
RPS6KA4	SACS	SERPINB13	SHQ1	SLX4	SMC3	SOX9	STAT5B
SYK	TCF3	TGFBR1	TNFRSF14	TRAF7	TTLL9	VTCN1	XPO1
YES1	ZNF750						

Table S2 Somatic gene variants of the primary tumor from the patient

Gene	Variant	AA change	Abundance	Variant type
TP53	c.861G>C	p.E287D	29.2%	Missense
TP53	c.853G>A	p.E285K	27.9%	Missense
ERBB2	c.2327G>T	p.G776V	20.7%	Missense
KDM6A	c.3790C>T	p.Q1264X	15.6%	Nonsense
KMT2D	c.12850C>T	p.Q1264X	9.4%	Nonsense
MLH1	c.12850C>T	p.Q1264X	7.6%	Nonsense
ERBB2	c.929C>T	p.S310F	6.9%	Missense
ARID1A	c.1669C>T	p.Q557X	6.0%	Nonsense
CFAP52	c.304G>C	p.E102Q	26.9%	Missense
ARHGAP23	c.3670G>A	p.E1224K	21.3%	Missense
FAM83C	c.544G>A	p.D182N	18.6%	Missense
CEP95	c.283G>C	p.E95Q	18.2%	Missense
RIPOR3	c.170C>A	p.S57Y	17.9%	Missense
SLC9A8	c.1030C>G	p.Q344E	17.6%	Missense
OTUD5	c.202C>G	p.Q68E	16.9%	Missense
C8orf88	c.65C>T	p.S22F	16.7%	Missense
EPB41L1	c.1225C>G	p.L409V	16.5%	Missense
FAM120C	c.1211C>T	p.S404L	15.2%	Missense
SENP3	c.1360G>C	p.E454Q	14.7%	Missense
SLC22A18	c.409C>T	p.Q137X	14.5%	Nonsense
SLC9A8	c.25G>T	p.E9X	14.5%	Nonsense
FAM8A1	c.280G>A	p.E94K	13.8%	Missense
CIPC	c.848C>A	p.S283X	13.6%	Nonsense
TTN	c.81424G>A	p.E27142K	13.4%	Missense
VPS53	c.379G>C	p.E127Q	13.3%	Missense
FBN2	c.5890G>C	p.E1964Q	13.2%	Missense
SLC12A5	c.1541C>G	p.S514W	12.8%	Missense
TMC5	c.2271C>G	p.I757M	12.7%	Missense
FAM120B	c.2303G>C	p.R768T	12.6%	Missense
APOBR	c.3151G>T	p.E1051X	12.5%	Nonsense
BICDL2	c.211C>T	p.L71F	12.4%	Missense
GGNBP2	c.1079C>T	p.S360L	12.3%	Missense
IL17RA	c.739G>C	p.E247Q	12.2%	Missense
MAP7D2	c.73G>A	p.G25R	11.7%	Missense
KIAA1522	c.2093C>T	p.S698F	11.3%	Missense
ZNF644	c.1408C>T	p.H470Y	11.3%	Missense
MUC2	c.2662C>T	p.Q888X	11.3%	Nonsense
HECTD4	c.7490C>T	p.S2497F	11.3%	Missense
NKD1	c.1088G>C	p.R363T	11.1%	Missense
DNAH8	c.4601A>G	p.N1534S	11.1%	Missense
CEP350	c.628G>A	p.E210K	11.0%	Missense
UBE2J2	c.208G>A	p.E70K	10.9%	Missense

TMOD4	c.850G>C	p.E284Q	10.9%	Missense
ARFRP1	c.562G>A	p.V188M	10.8%	Missense
FAM126B	c.502C>T	p.Q168X	10.3%	Nonsense
PTCH2	c.1747G>C	p.E583Q	10.2%	Missense
DHX8	c.2259G>C	p.E753D	10.2%	Missense
CEP97	c.2356G>A	p.D786N	10.2%	Missense
COPS2	c.861G>A	p.M287I	10.1%	Missense
CTH	c.585C>G	p.F195L	10.0%	Missense
VPS13C	c.3856C>A	p.P1286T	10.0%	Missense
OXTR	c.215C>G	p.S72W	9.9%	Missense
TNRC6B	c.3454G>A	p.E1152K	9.8%	Missense
SLC13A1	c.1256C>G	p.S419C	9.8%	Missense
TNFRSF1A	c.935G>C	p.R312T	9.7%	Missense
CLMN	c.1207C>T	p.P403S	9.7%	Missense
RNASEL	c.1804G>C	p.E602Q	9.4%	Missense
FIZ1	c.850G>A	p.D284N	9.4%	Missense
CLIP1	c.2111C>T	p.S704L	9.3%	Missense
DDX19A	c.348G>C	p.K116N	9.3%	Missense
P4HB	c.1252G>T	p.E418X	9.3%	Nonsense
CCNG2	c.223G>A	p.E75K	9.3%	Missense
MUC2	c.2489C>T	p.S830F	9.2%	Missense
MFAP1	c.361G>C	p.E121Q	9.2%	Missense
RFX1	c.1753G>C	p.D585H	9.2%	Missense
SHC3	c.341C>T	p.S114L	9.1%	Missense
ZNF497	c.546G>C	p.E182D	9.0%	Missense
ASAP3	c.950G>A	p.R317Q	8.9%	Missense
ZPBP	c.617C>G	p.S206X	8.8%	Nonsense
PTPN9	c.247C>T	p.H83Y	8.7%	Missense
BBS9	c.1804C>T	p.Q602X	8.7%	Nonsense
MELK	c.448G>C	p.D150H	8.7%	Missense
NOMO3	c.1008G>C	p.L336F	8.6%	Missense
PHLDB3	c.427G>A	p.E143K	8.6%	Missense
FAP	c.841C>T	p.P281S	8.6%	Missense
PPARG	c.854G>A	p.C285Y	8.5%	Missense
SOX12	c.56G>A	p.G19E	8.4%	Missense
AK9	c.3751G>A	p.E1251K	8.3%	Missense
VPS11	c.2109G>C	p.Q703H	8.2%	Missense
TBC1D16	c.2224G>C	p.E742Q	8.2%	Missense
KLRG1	c.41C>T	p.T14M	8.1%	Missense
P2RX4	c.1021G>A	p.D341N	8.1%	Missense
GSTM3	c.10G>A	p.E4K	8.0%	Missense
BRWD1	c.1457C>G	p.S486C	8.0%	Missense
CD40	c.530C>T	p.A177V	7.9%	Missense
GFI1B	c.972G>C	p.E324D	7.9%	Missense

MAGEA4	c.467C>T	p.S156F	7.8%	Missense
MCMBP	c.427G>C	p.E143Q	7.7%	Missense
DNAH5	c.5434C>G	p.Q1812E	7.6%	Missense
ZFYVE16	c.206C>G	p.S69X	7.6%	Nonsense
LAMA2	c.2614G>A	p.D872N	7.6%	Missense
IRX3	c.586C>T	p.R196C	7.5%	Missense
XAB2	c.2149G>A	p.E717K	7.5%	Missense
GLS	c.920G>A	p.R307Q	7.5%	Missense
NUMB	c.764C>T	p.A255V	7.3%	Missense
TM2D3	c.331C>G	p.Q111E	7.3%	Missense
PIK3C2B	c.1215C>G	p.I405M	7.2%	Missense
